# 4,10-Diall­yloxy-1,2,3,6b,7,8,9,12b-octa­hydro­perylene

**DOI:** 10.1107/S160053680905243X

**Published:** 2009-12-09

**Authors:** Terrill D. Smith, Mathew P. D. Mahindaratne, Mark A. Penick, George R. Negrete, Lee M. Daniels, Edward R. T. Tiekink

**Affiliations:** a Department of Chemistry, University of Texas at San Antonio, One UTSA Circle, San Antonio, Texas 78249-0698, USA; b Rigaku Americas Corporation, 9009 New Trails Drive, The Woodlands, Texas 77381, USA; c Department of Chemistry, University of Malaya, 50603 Kuala Lumpur, Malaysia

## Abstract

In the title compound, C_26_H_28_O_2_, the central atoms are coplanar, with the –CH_2_—CH_2_– links of the cyclo­hexene groups lying to either side of the plane and with the diall­yloxy residues twisted out of this plane [C—C—O—C torsion angles = 16.6 (3) and −13.9 (3)°]. In the crystal structure, mol­ecules are connected into chains propagating in [100] *via* C—H⋯π inter­actions.

## Related literature

For the preparation of oxygenated perylenes and their use as photosensitizing organic dyes in solar harvesting techniques, see: Penick *et al.* (2008 ▶).
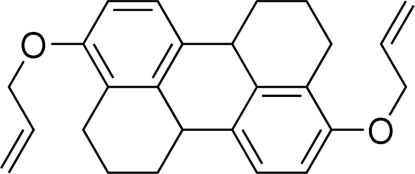


## Experimental

### 

#### Crystal data

C_26_H_28_O_2_
*M* *_r_* = 372.48
Monoclinic, 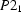

*a* = 4.5883 (1) Å
*b* = 14.9171 (3) Å
*c* = 13.9203 (3) Å
β = 95.153 (1)°
*V* = 948.92 (3) Å^3^
*Z* = 2
Cu *K*α radiation
μ = 0.63 mm^−1^
*T* = 100 K
0.50 × 0.19 × 0.11 mm

#### Data collection

Rigaku RAXIS RAPID diffractometer
Absorption correction: multi-scan (*ABSCOR*; Higashi, 1995 ▶) *T*_min_ = 0.745, *T*_max_ = 0.935
8967 measured reflections
3243 independent reflections
2616 reflections with *I* > 2σ(*I*)
*R*_int_ = 0.039

#### Refinement

*R*[*F* ^2^ > 2σ(*F* ^2^)] = 0.036
*wR*(*F* ^2^) = 0.104
*S* = 1.26
3243 reflections
254 parameters
1 restraint
H-atom parameters constrained
Δρ_max_ = 0.21 e Å^−3^
Δρ_min_ = −0.19 e Å^−3^

### 

Data collection: *CrystalClear* (Rigaku/MSC, 2005 ▶); cell refinement: *PROCESS-AUTO* (Rigaku, 1998 ▶); data reduction: *PROCESS-AUTO*; program(s) used to solve structure: *SHELXS97* (Sheldrick, 2008 ▶); program(s) used to refine structure: *SHELXL97* (Sheldrick, 2008 ▶); molecular graphics: *DIAMOND* (Brandenburg, 2006 ▶); software used to prepare material for publication: *publCIF* (Westrip, 2009 ▶).

## Supplementary Material

Crystal structure: contains datablocks global, I. DOI: 10.1107/S160053680905243X/hb5266sup1.cif

Structure factors: contains datablocks I. DOI: 10.1107/S160053680905243X/hb5266Isup2.hkl

Additional supplementary materials:  crystallographic information; 3D view; checkCIF report

## Figures and Tables

**Table 1:** Hydrogen-bond geometry (Å, °)

*D*—H⋯*A*	*D*—H	H⋯*A*	*D*⋯*A*	*D*—H⋯*A*
C13—H13⋯*Cg*1^i^	1.00	2.78	3.671 (3)	148
C20—H20⋯*Cg*4^ii^	1.00	2.82	3.702 (3)	148

Symmetry codes: (i) 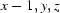; (ii) 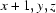. *Cg*1 and *Cg*4 are the centroids of the C4–C9 and C14–C19 rings, respectively.

**Fig. 1.**
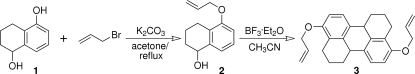
Reaction scheme.

**Fig. 2.**
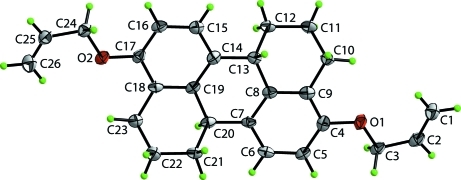
Molecular structure of the title compound, showing displacement ellipsoids at the 70% probability level.

**Fig. 3.**
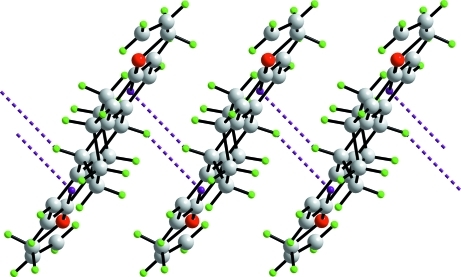
The supramolecular chains in the title compound aligned along [1 0 0] sustained by C–H···π interactions which are represented by purple dashed lines. Color code: O, red; C, grey; and H, green.

**Fig. 4.**
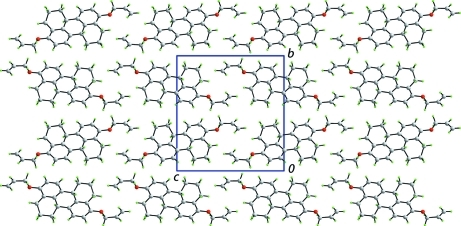
A view in projection down the a axis of the crystal packing in the title compound. Color code: O, red; C, grey; and H, green.

## supplementary crystallographic information

### Comment

Our laboratory has been investigating the preparation of oxygenated perylenes and their use as photosensitizing organic dyes in solar harvesting techniques (Penick *et al.*, 2008). The commercially available starting material 1,2,3,4-tetrahydronaphthalen-1,5-diol (1), Fig. 1, was allylated selectively at the phenolic-OH using equimolar amounts of allyl bromide and K_2_CO_3_ in refluxing acetone. The resulting 5-allyloxy-1,2,3,4-tetrahydronaphthalen-1-ol (2), Fig. 2, was subjected to tandem Friedel-Crafts annulation (Penick *et al.*, 2008) in acetonitrile at room temperature using BF_3_ as the Lewis acid catalyst. The product (3), Fig. 1, precipitated and was purified by trituration with acetone to produce a white solid. Product 3 was crystallized using a slow evaporation method from chloroform to obtain single crystals for spectroscopic and X-ray crystallographic analysis.

The molecular structure of the title compound, Fig. 2, features an essentially planar core. Thus, the maximum deviations from the least-squares plane through the benzene rings (atoms C4–C9 and C14–C19) as well as the *sp*^3^ O1, O2, C10, C13, C20, and C23 atoms are 0.0509 (23) Å for atom C10 and -0.0389 (21) Å for atom C8 (r.m.s. = 0.0242 Å). The C11 and C12, and C21 and C22 atoms of the cyclohexene rings lie to either side of this plane. The planarity in the molecule does not extend to the terminal diallyloxy residues as seen in the magnitudes of the C5–C4–O1–O3 and C16–C17–O2–C24 torsion angles of 16.6 (3) and -13.9 (3) °, respectively.

The most prominent feature of the crystal packing is the presence of C–H···π interactions that link molecules into supramolecular chains along [1 0 0], Fig. 3. The geometric parameters associated with these interactions are C13–H13···*Cg*(C4–C9)^i^ = 2.78 Å, C13···*Cg*(C4–C9)^i^ = 3.671 (3) Å with an angle of 148° at H13 for symmetry operation i: -1 + *x*, *y*, *z*; and C20–H20···*Cg*(C14–C19)^ii^ = 2.82 Å, C20···*Cg*(C14–C19)^ii^ = 3.702 (3) Å with an angle of 148° at H20 for ii: 1 + *x*, *y*, *z*. Supramolecular chains are consolidated in the crystal structure by hydrophobic interactions, Fig. 4.

### Experimental

Referring to Fig. 1, diol 1 was converted to allyl aryl ether 2*via* conventional phenolic alkylation (allyl bromide/K_2_CO_3_/acetone). Octahydroperylene 3 was prepared upon treatment of allyl aryl ether 2 (173 mg, 0.85 mmol) in acetonitrile (5 ml) with BF_3_.Et_2_O (0.5 ml) by dropwise addition over one minute and the mixture was stirred for 43 h at room temperature. An off-white solid was collected by filtration, triturated with acetone, and dried under vacuum. The solid was crystallized by slowly evaporating its CHCl_3_ solution to yield off-white small rods (84 mg, 53%), *M*. pt. = 458–461 K; ^1^H NMR (CDCl_3_, 500 MHz): δ 1.51 (tt, J = 12.2, 8.3 Hz, 2H), 1.69–1.79 (m, 2H), 2.00–2.10 (m, 2H), 2.53 (dt, J = 16.1, 7.8 Hz, 2H), 2.51–2.58 (m, 2H), 3.15 (ddd, J = 16.1, 6.8, 5.4 Hz, 2H), 3.73 (dd, J = 11.7, 4.4 Hz, 2H), 4.51–4.59 (m, two ABX patterns, 4H), 5.27 (dq, J = 10.8, 1.5 Hz, 2H), 5.44 (dq, J = 17.1, 1.5 Hz, 2H), 6.95 (ddt, J = 17.1, 10.8, 5.4 Hz, 2H), 6.82 (d, J = 7.8 Hz, 2H), 7.23 (d, J = 8.3 Hz, 2H) p.p.m. ^13^C NMR (CDCl_3_, 125 MHz): δ 20.9 (*t*), 21.19 (*t*), 29.89 (*t*), 36.09 (*d*), 69.29 (*t*), 110.1 (*d*), 116.9 (dd), 124.5 (*d*), 126.7 (*s*), 128.2 (*s*), 133.9 (*d*), 136.9 (*s*), 153.5 (*s*) p.p.m. IR (ν_max_, cm^-1^): 2933, 2913, 2857, 1486, 1464, 1420, 1258, 1071, 1033, 997. 924, 797. MS (APCI, m/*z*): 373.4 (52, *M*^+^+1), 372.4 (34, *M*^+^), 371.4 (100, *M*^+^-1), 330.4 (33, *M*^+^-C_3_H_6_).

### Refinement

The H atoms were geometrically placed (C—H = 0.95–1.00 Å) and refined as riding with *U*_iso_(H) = 1.2*U*_eq_(parent atom). The structure was refined as a racemic twin precluding the determination of absolute structure.

### Crystal data

**Table:**

C_26_H_28_O_2_	*F*(000) = 400
*M**_r_* = 372.48	*D*_x_ = 1.304 Mg m^−^^3^
Monoclinic, *P*2_1_	Cu *K*α radiation, λ = 1.54187 Å
Hall symbol: P 2yb	Cell parameters from 8127 reflections
*a* = 4.5883 (1) Å	θ = 6.7–70.1°
*b* = 14.9171 (3) Å	µ = 0.63 mm^−^^1^
*c* = 13.9203 (3) Å	*T* = 100 K
β = 95.153 (1)°	Prism, colourless
*V* = 948.92 (3) Å^3^	0.50 × 0.19 × 0.11 mm
*Z* = 2	

### Data collection

**Table:**

Rigaku RAXIS RAPID diffractometer	3243 independent reflections
Radiation source: fine-focus sealed tube	2616 reflections with *I* > 2σ(*I*)
graphite	*R*_int_ = 0.039
Profile data from ω scans	θ_max_ = 70.0°, θ_min_ = 6.7°
Absorption correction: multi-scan (*ABSCOR*; Higashi, 1995)	*h* = −4→5
*T*_min_ = 0.745, *T*_max_ = 0.935	*k* = −18→17
8967 measured reflections	*l* = −16→16

### Refinement

**Table:**

Refinement on *F*^2^	Primary atom site location: structure-invariant direct methods
Least-squares matrix: full	Secondary atom site location: difference Fourier map
*R*[*F*^2^ > 2σ(*F*^2^)] = 0.036	Hydrogen site location: inferred from neighbouring sites
*wR*(*F*^2^) = 0.104	H-atom parameters constrained
*S* = 1.26	*w* = 1/[σ^2^(*F*_o_^2^) + (0.0392*P*)^2^ + 0.0986*P*] where *P* = (*F*_o_^2^ + 2*F*_c_^2^)/3
3243 reflections	(Δ/σ)_max_ < 0.001
254 parameters	Δρ_max_ = 0.21 e Å^−^^3^
1 restraint	Δρ_min_ = −0.19 e Å^−^^3^

### Special details

**Table:**

Geometry. All s.u.'s (except the s.u. in the dihedral angle between two l.s. planes) are estimated using the full covariance matrix. The cell s.u.'s are taken into account individually in the estimation of s.u.'s in distances, angles and torsion angles; correlations between s.u.'s in cell parameters are only used when they are defined by crystal symmetry. An approximate (isotropic) treatment of cell s.u.'s is used for estimating s.u.'s involving l.s. planes.
Refinement. Refinement of *F*^2^ against ALL reflections. The weighted *R*-factor *wR* and goodness of fit *S* are based on *F*^2^, conventional *R*-factors *R* are based on *F*, with *F* set to zero for negative *F*^2^. The threshold expression of *F*^2^ > 2σ(*F*^2^) is used only for calculating *R*-factors(gt) *etc*. and is not relevant to the choice of reflections for refinement. *R*-factors based on *F*^2^ are statistically about twice as large as those based on *F*, and *R*- factors based on ALL data will be even larger.

### Fractional atomic coordinates and isotropic or equivalent isotropic displacement parameters (Å^2^)

**Table:**

	*x*	*y*	*z*	*U*_iso_*/*U*_eq_	
O1	1.0758 (4)	0.14440 (11)	1.29479 (11)	0.0205 (4)	
O2	0.3720 (4)	0.35698 (11)	0.62545 (11)	0.0209 (4)	
C1	1.1560 (6)	0.14679 (19)	1.49090 (18)	0.0257 (6)	
H1A	1.0022	0.1804	1.4579	0.031*	
H1B	1.1811	0.1471	1.5594	0.031*	
C2	1.3344 (6)	0.09973 (17)	1.44171 (18)	0.0215 (6)	
H2	1.4851	0.0671	1.4776	0.026*	
C3	1.3204 (6)	0.09320 (16)	1.33434 (16)	0.0185 (6)	
H3A	1.5030	0.1168	1.3110	0.022*	
H3B	1.2979	0.0298	1.3140	0.022*	
C4	1.0339 (5)	0.15331 (16)	1.19565 (16)	0.0173 (6)	
C5	1.1684 (5)	0.09822 (16)	1.13154 (17)	0.0182 (6)	
H5	1.2992	0.0521	1.1547	0.022*	
C6	1.1068 (5)	0.11230 (16)	1.03333 (17)	0.0179 (5)	
H6	1.2013	0.0758	0.9896	0.022*	
C7	0.9124 (5)	0.17770 (15)	0.99632 (17)	0.0146 (5)	
C8	0.7818 (5)	0.23265 (14)	1.06198 (17)	0.0153 (5)	
C9	0.8430 (5)	0.22077 (15)	1.16185 (18)	0.0161 (5)	
C10	0.7069 (6)	0.28600 (17)	1.22696 (16)	0.0179 (6)	
H10A	0.7947	0.2781	1.2940	0.021*	
H10B	0.4943	0.2742	1.2257	0.021*	
C11	0.7571 (6)	0.38257 (16)	1.19372 (17)	0.0226 (6)	
H11A	0.9626	0.3997	1.2129	0.027*	
H11B	0.6291	0.4235	1.2270	0.027*	
C12	0.6945 (6)	0.39485 (15)	1.08386 (16)	0.0188 (5)	
H12A	0.8768	0.4137	1.0566	0.023*	
H12B	0.5487	0.4433	1.0713	0.023*	
C13	0.5781 (6)	0.30864 (14)	1.03188 (16)	0.0148 (6)	
H13	0.3844	0.2946	1.0560	0.018*	
C14	0.5266 (5)	0.32240 (15)	0.92412 (18)	0.0153 (6)	
C15	0.3363 (5)	0.38949 (16)	0.88675 (17)	0.0182 (6)	
H15	0.2430	0.4269	0.9300	0.022*	
C16	0.2800 (5)	0.40290 (16)	0.78830 (17)	0.0187 (6)	
H16	0.1519	0.4496	0.7648	0.022*	
C17	0.4106 (5)	0.34823 (15)	0.72429 (16)	0.0158 (5)	
C18	0.5998 (5)	0.27982 (16)	0.75910 (16)	0.0160 (5)	
C19	0.6584 (5)	0.26758 (15)	0.85844 (16)	0.0134 (5)	
C20	0.8599 (6)	0.19078 (16)	0.88847 (17)	0.0168 (6)	
H20	1.0537	0.2044	0.8642	0.020*	
C21	0.7403 (6)	0.10527 (16)	0.83550 (16)	0.0199 (6)	
H21A	0.8787	0.0552	0.8506	0.024*	
H21B	0.5510	0.0888	0.8596	0.024*	
C22	0.6961 (6)	0.11772 (17)	0.72538 (17)	0.0242 (6)	
H22A	0.8354	0.0786	0.6947	0.029*	
H22B	0.4955	0.0985	0.7021	0.029*	
C23	0.7414 (6)	0.21513 (15)	0.69392 (17)	0.0189 (6)	
H23A	0.9535	0.2280	0.6960	0.023*	
H23B	0.6556	0.2233	0.6267	0.023*	
C24	0.1371 (6)	0.41273 (16)	0.58588 (17)	0.0197 (6)	
H24A	0.1660	0.4749	0.6099	0.024*	
H24B	−0.0512	0.3903	0.6060	0.024*	
C25	0.1309 (6)	0.41135 (16)	0.47868 (17)	0.0216 (6)	
H25	−0.0200	0.4447	0.4437	0.026*	
C26	0.3135 (6)	0.36865 (18)	0.42822 (18)	0.0272 (6)	
H26A	0.4681	0.3344	0.4600	0.033*	
H26B	0.2917	0.3718	0.3598	0.033*	

### Atomic displacement parameters (Å^2^)

**Table:**

	*U*^11^	*U*^22^	*U*^33^	*U*^12^	*U*^13^	*U*^23^
O1	0.0198 (10)	0.0258 (10)	0.0154 (8)	0.0072 (8)	−0.0006 (7)	0.0045 (7)
O2	0.0214 (11)	0.0219 (10)	0.0190 (9)	0.0062 (8)	−0.0009 (8)	0.0030 (8)
C1	0.0292 (17)	0.0258 (14)	0.0209 (13)	0.0025 (12)	−0.0032 (11)	0.0019 (11)
C2	0.0180 (15)	0.0208 (13)	0.0246 (14)	0.0024 (11)	−0.0037 (11)	0.0041 (11)
C3	0.0169 (15)	0.0165 (12)	0.0213 (14)	0.0006 (10)	−0.0031 (11)	0.0025 (11)
C4	0.0160 (15)	0.0191 (13)	0.0165 (12)	−0.0034 (11)	−0.0006 (11)	0.0021 (11)
C5	0.0178 (15)	0.0157 (12)	0.0207 (13)	0.0033 (11)	0.0001 (11)	0.0027 (10)
C6	0.0175 (15)	0.0133 (12)	0.0233 (13)	0.0004 (10)	0.0033 (10)	−0.0049 (10)
C7	0.0140 (15)	0.0111 (12)	0.0185 (12)	−0.0015 (9)	−0.0010 (10)	−0.0023 (10)
C8	0.0130 (14)	0.0132 (13)	0.0194 (13)	−0.0012 (10)	−0.0004 (11)	−0.0008 (10)
C9	0.0121 (15)	0.0144 (13)	0.0219 (13)	−0.0025 (10)	0.0023 (11)	−0.0005 (10)
C10	0.0202 (15)	0.0156 (13)	0.0176 (13)	0.0019 (11)	0.0001 (11)	−0.0031 (10)
C11	0.0311 (17)	0.0133 (13)	0.0229 (14)	−0.0047 (11)	−0.0001 (12)	−0.0038 (10)
C12	0.0231 (15)	0.0118 (12)	0.0213 (12)	−0.0007 (10)	0.0013 (10)	−0.0018 (9)
C13	0.0142 (15)	0.0135 (13)	0.0172 (13)	−0.0015 (9)	0.0041 (10)	−0.0013 (9)
C14	0.0127 (15)	0.0148 (12)	0.0186 (12)	−0.0029 (10)	0.0023 (10)	0.0017 (10)
C15	0.0181 (15)	0.0156 (12)	0.0212 (13)	0.0008 (10)	0.0030 (11)	0.0001 (10)
C16	0.0172 (15)	0.0155 (13)	0.0228 (14)	0.0027 (10)	−0.0008 (11)	0.0017 (11)
C17	0.0161 (16)	0.0144 (13)	0.0165 (12)	−0.0025 (10)	−0.0011 (10)	0.0024 (10)
C18	0.0140 (14)	0.0130 (12)	0.0209 (12)	−0.0004 (10)	0.0018 (10)	−0.0007 (10)
C19	0.0111 (13)	0.0099 (12)	0.0190 (12)	−0.0054 (10)	−0.0002 (10)	0.0016 (10)
C20	0.0144 (15)	0.0138 (13)	0.0223 (14)	−0.0007 (10)	0.0016 (11)	0.0001 (10)
C21	0.0262 (16)	0.0121 (12)	0.0213 (13)	−0.0006 (11)	0.0015 (11)	−0.0030 (10)
C22	0.0333 (17)	0.0180 (14)	0.0205 (13)	0.0015 (11)	−0.0022 (12)	−0.0020 (10)
C23	0.0215 (16)	0.0155 (13)	0.0194 (13)	0.0022 (10)	0.0011 (11)	0.0001 (10)
C24	0.0205 (16)	0.0178 (12)	0.0203 (13)	0.0032 (11)	−0.0006 (11)	0.0026 (11)
C25	0.0254 (17)	0.0178 (13)	0.0208 (13)	−0.0006 (11)	−0.0022 (11)	0.0022 (10)
C26	0.0311 (17)	0.0282 (15)	0.0210 (13)	0.0008 (12)	−0.0042 (11)	−0.0003 (11)

### Geometric parameters (Å, °)

**Table:**

O1—C4	1.383 (3)	C12—H12B	0.9900
O1—C3	1.427 (3)	C13—C14	1.511 (3)
O2—C17	1.378 (3)	C13—H13	1.0000
O2—C24	1.432 (3)	C14—C15	1.398 (3)
C1—C2	1.316 (3)	C14—C19	1.403 (3)
C1—H1A	0.9500	C15—C16	1.386 (3)
C1—H1B	0.9500	C15—H15	0.9500
C2—C3	1.493 (3)	C16—C17	1.383 (3)
C2—H2	0.9500	C16—H16	0.9500
C3—H3A	0.9900	C17—C18	1.398 (3)
C3—H3B	0.9900	C18—C19	1.397 (3)
C4—C9	1.388 (3)	C18—C23	1.511 (3)
C4—C5	1.397 (3)	C19—C20	1.508 (3)
C5—C6	1.387 (3)	C20—C21	1.549 (3)
C5—H5	0.9500	C20—H20	1.0000
C6—C7	1.389 (3)	C21—C22	1.539 (3)
C6—H6	0.9500	C21—H21A	0.9900
C7—C8	1.402 (3)	C21—H21B	0.9900
C7—C20	1.512 (3)	C22—C23	1.537 (3)
C8—C9	1.405 (3)	C22—H22A	0.9900
C8—C13	1.504 (3)	C22—H22B	0.9900
C9—C10	1.503 (3)	C23—H23A	0.9900
C10—C11	1.537 (3)	C23—H23B	0.9900
C10—H10A	0.9900	C24—C25	1.490 (3)
C10—H10B	0.9900	C24—H24A	0.9900
C11—C12	1.541 (3)	C24—H24B	0.9900
C11—H11A	0.9900	C25—C26	1.306 (3)
C11—H11B	0.9900	C25—H25	0.9500
C12—C13	1.547 (3)	C26—H26A	0.9500
C12—H12A	0.9900	C26—H26B	0.9500
			
C4—O1—C3	118.08 (18)	C12—C13—H13	107.3
C17—O2—C24	117.67 (18)	C15—C14—C19	117.8 (2)
C2—C1—H1A	120.0	C15—C14—C13	120.2 (2)
C2—C1—H1B	120.0	C19—C14—C13	122.0 (2)
H1A—C1—H1B	120.0	C16—C15—C14	121.7 (2)
C1—C2—C3	125.6 (2)	C16—C15—H15	119.1
C1—C2—H2	117.2	C14—C15—H15	119.1
C3—C2—H2	117.2	C17—C16—C15	119.9 (2)
O1—C3—C2	108.2 (2)	C17—C16—H16	120.0
O1—C3—H3A	110.1	C15—C16—H16	120.0
C2—C3—H3A	110.1	O2—C17—C16	124.3 (2)
O1—C3—H3B	110.1	O2—C17—C18	115.8 (2)
C2—C3—H3B	110.1	C16—C17—C18	119.9 (2)
H3A—C3—H3B	108.4	C19—C18—C17	119.8 (2)
O1—C4—C9	115.8 (2)	C19—C18—C23	117.1 (2)
O1—C4—C5	123.5 (2)	C17—C18—C23	123.0 (2)
C9—C4—C5	120.8 (2)	C18—C19—C14	120.9 (2)
C6—C5—C4	118.5 (2)	C18—C19—C20	115.6 (2)
C6—C5—H5	120.7	C14—C19—C20	123.5 (2)
C4—C5—H5	120.7	C19—C20—C7	114.37 (19)
C5—C6—C7	122.6 (2)	C19—C20—C21	108.16 (19)
C5—C6—H6	118.7	C7—C20—C21	112.61 (18)
C7—C6—H6	118.7	C19—C20—H20	107.1
C6—C7—C8	117.8 (2)	C7—C20—H20	107.1
C6—C7—C20	119.9 (2)	C21—C20—H20	107.1
C8—C7—C20	122.2 (2)	C22—C21—C20	112.59 (18)
C7—C8—C9	120.8 (2)	C22—C21—H21A	109.1
C7—C8—C13	123.4 (2)	C20—C21—H21A	109.1
C9—C8—C13	115.8 (2)	C22—C21—H21B	109.1
C4—C9—C8	119.4 (2)	C20—C21—H21B	109.1
C4—C9—C10	123.3 (2)	H21A—C21—H21B	107.8
C8—C9—C10	117.3 (2)	C23—C22—C21	112.9 (2)
C9—C10—C11	110.1 (2)	C23—C22—H22A	109.0
C9—C10—H10A	109.6	C21—C22—H22A	109.0
C11—C10—H10A	109.6	C23—C22—H22B	109.0
C9—C10—H10B	109.6	C21—C22—H22B	109.0
C11—C10—H10B	109.6	H22A—C22—H22B	107.8
H10A—C10—H10B	108.2	C18—C23—C22	110.9 (2)
C10—C11—C12	112.98 (19)	C18—C23—H23A	109.5
C10—C11—H11A	109.0	C22—C23—H23A	109.5
C12—C11—H11A	109.0	C18—C23—H23B	109.5
C10—C11—H11B	109.0	C22—C23—H23B	109.5
C12—C11—H11B	109.0	H23A—C23—H23B	108.1
H11A—C11—H11B	107.8	O2—C24—C25	108.87 (19)
C11—C12—C13	113.02 (18)	O2—C24—H24A	109.9
C11—C12—H12A	109.0	C25—C24—H24A	109.9
C13—C12—H12A	109.0	O2—C24—H24B	109.9
C11—C12—H12B	109.0	C25—C24—H24B	109.9
C13—C12—H12B	109.0	H24A—C24—H24B	108.3
H12A—C12—H12B	107.8	C26—C25—C24	126.1 (2)
C8—C13—C14	114.61 (19)	C26—C25—H25	116.9
C8—C13—C12	108.44 (19)	C24—C25—H25	116.9
C14—C13—C12	111.59 (18)	C25—C26—H26A	120.0
C8—C13—H13	107.3	C25—C26—H26B	120.0
C14—C13—H13	107.3	H26A—C26—H26B	120.0
			
C4—O1—C3—C2	175.20 (19)	C19—C14—C15—C16	−0.7 (3)
C1—C2—C3—O1	−1.7 (4)	C13—C14—C15—C16	−178.8 (2)
C3—O1—C4—C9	−164.3 (2)	C14—C15—C16—C17	0.9 (4)
C3—O1—C4—C5	16.6 (3)	C24—O2—C17—C16	−13.9 (3)
O1—C4—C5—C6	179.0 (2)	C24—O2—C17—C18	167.3 (2)
C9—C4—C5—C6	−0.1 (3)	C15—C16—C17—O2	−179.0 (2)
C4—C5—C6—C7	−1.3 (4)	C15—C16—C17—C18	−0.2 (4)
C5—C6—C7—C8	1.8 (4)	O2—C17—C18—C19	178.24 (19)
C5—C6—C7—C20	178.7 (2)	C16—C17—C18—C19	−0.7 (3)
C6—C7—C8—C9	−0.9 (3)	O2—C17—C18—C23	−3.5 (3)
C20—C7—C8—C9	−177.7 (2)	C16—C17—C18—C23	177.5 (2)
C6—C7—C8—C13	177.2 (2)	C17—C18—C19—C14	0.9 (3)
C20—C7—C8—C13	0.3 (3)	C23—C18—C19—C14	−177.4 (2)
O1—C4—C9—C8	−178.18 (19)	C17—C18—C19—C20	178.7 (2)
C5—C4—C9—C8	0.9 (3)	C23—C18—C19—C20	0.4 (3)
O1—C4—C9—C10	5.0 (3)	C15—C14—C19—C18	−0.2 (3)
C5—C4—C9—C10	−175.9 (2)	C13—C14—C19—C18	177.9 (2)
C7—C8—C9—C4	−0.4 (3)	C15—C14—C19—C20	−177.9 (2)
C13—C8—C9—C4	−178.7 (2)	C13—C14—C19—C20	0.2 (3)
C7—C8—C9—C10	176.6 (2)	C18—C19—C20—C7	−178.4 (2)
C13—C8—C9—C10	−1.6 (3)	C14—C19—C20—C7	−0.6 (3)
C4—C9—C10—C11	128.2 (2)	C18—C19—C20—C21	−52.0 (3)
C8—C9—C10—C11	−48.6 (3)	C14—C19—C20—C21	125.8 (2)
C9—C10—C11—C12	45.6 (3)	C6—C7—C20—C19	−176.4 (2)
C10—C11—C12—C13	3.3 (3)	C8—C7—C20—C19	0.3 (3)
C7—C8—C13—C14	−0.7 (3)	C6—C7—C20—C21	59.6 (3)
C9—C8—C13—C14	177.4 (2)	C8—C7—C20—C21	−123.7 (2)
C7—C8—C13—C12	−126.1 (2)	C19—C20—C21—C22	54.4 (3)
C9—C8—C13—C12	52.0 (3)	C7—C20—C21—C22	−178.2 (2)
C11—C12—C13—C8	−51.3 (3)	C20—C21—C22—C23	−7.6 (3)
C11—C12—C13—C14	−178.5 (2)	C19—C18—C23—C22	48.7 (3)
C8—C13—C14—C15	178.5 (2)	C17—C18—C23—C22	−129.6 (2)
C12—C13—C14—C15	−57.8 (3)	C21—C22—C23—C18	−42.6 (3)
C8—C13—C14—C19	0.5 (3)	C17—O2—C24—C25	−178.87 (19)
C12—C13—C14—C19	124.2 (2)	O2—C24—C25—C26	−1.8 (4)

### Hydrogen-bond geometry (Å, °)

**Table:**

Cg1 and Cg4 are the centroids of the C4–C9 and C14–C19 rings, respectively.

**Table:**

*D*—H···*A*	*D*—H	H···*A*	*D*···*A*	*D*—H···*A*
C13—H13···Cg1^i^	1.00	2.78	3.671 (3)	148
C20—H20···Cg4^ii^	1.00	2.82	3.702 (3)	148

Symmetry codes: (i) *x*−1, *y*, *z*; (ii) *x*+1, *y*, *z*.

## References

[bb1] Brandenburg, K. (2006). *DIAMOND* Crystal Impact GbR, Bonn, Germany.

[bb2] Higashi, T. (1995). *ABSCOR* Rigaku Corporation, Tokyo, Japan.

[bb3] Penick, M. A., Mahindaratne, M. P. D., Gutierrez, R. D., Smith, T. D., Tiekink, E. R. T. & Negrete, G. R. (2008). *J. Org. Chem.* **73**, 6378–6381. 10.1021/jo800558c PMC4780845 18630879

[bb4] Rigaku (1998). *PROCESS-AUTO* Rigaku Corporation, Tokyo, Japan.

[bb5] Rigaku/MSC (2005). *CrystalClear* Rigaku/MSC Inc., The Woodlands, Texas, USA.

[bb6] Sheldrick, G. M. (2008). *Acta Cryst.* A**64**, 112–122. 10.1107/S0108767307043930 18156677

[bb7] Westrip, S. P. (2009). *publCIF* In preparation.

